# Incongruence between residential uses and perceptions of fertilizers and deicers

**DOI:** 10.1371/journal.pone.0306550

**Published:** 2024-07-19

**Authors:** Heather D. Craska, Amélie Y. Davis

**Affiliations:** 1 Department of Natural Resources and Environmental Sciences, University of Illinois at Urbana-Champaign, Urbana, Illinois, United States of America; 2 Department of Economics and Geosciences, United States Air Force Academy, Colorado Springs, Colorado, United States of America; Universidad Autonoma de Chihuahua, MEXICO

## Abstract

Fertilizers and deicers are common materials for property maintenance in the Midwest, however, their application contributes to negative environmental impacts when applied incorrectly. While fertilizer use is well researched, deicer use on private properties is not. This research aims to ascertain whether patterns of fertilizer use are different from those of deicer use in Hamilton County, Ohio, and determine what factors influence a resident’s decision to use these materials. Survey data were collected from 110 single-family households (38.9% response rate). Respondents are motivated by property appearance to apply fertilizers. Deicer use stems from safety concerns. Respondents were significantly more likely to consider the environmental impact of fertilizers than deicers. Respondents felt that using deicers is a more neighborly practice while using fertilizers reflects more positively on them in their neighborhood. This information can be used to develop outreach programs to reduce the environmental impacts of fertilizers and deicers.

## Introduction

Two common practices of residential property maintenance include the application of fertilizers to lawns and gardens, as well as treating sidewalks and driveways with deicers. While safe and responsible methods of application for fertilizers and deicers exist, many residential landowners make mistakes or disregard application recommendations and apply fertilizers and deicers in larger quantities than needed [1, 2]. Excess fertilizers and deicers can have negative impacts on the environment and human health [3, 4]. Previous research has demonstrated that there are a multitude of social, educational, and normative determinants contributing to the issue of overfertilization [5–10]. However, determinants of deicer use have not been studied with nearly as much depth as fertilizers. Therefore, it is unclear if the determinants of deicer use are similar to those for fertilizers. We first summarize the environmental effects of fertilizers and deicers, then the uses and perceptions of fertilizers and deicers. Finally, research questions and methods are introduced to explore residential use and perceptions of fertilizers and deicers and compare them.

### Effects of deicers

The deicing materials applied to roads, sidewalks, and driveways to prevent and melt ice also contribute to the degradation of the environment. Various types of deicers can be applied in residential or commercial settings including chloride-based, acetate-based, and carbohydrates [11]. However, chloride-based deicers, specifically sodium chloride (NaCl), are the more prevalent of the ice control options [12–14]. These deicing chemicals often accumulate in soils, water bodies, and vegetation because they are highly mobile and are not biodegradable [13]. Rock salt deicers will disperse through the surrounding environment via snowmelt runoff, being splashed into roadside water or soil by moving traffic, or by the removal and displacement of salt-laden ice and snow [12]. Studies from Struzeski [15] and Godwin et al. [16] focused on the effects of rock salts in Lake Erie and the Mohawk River Basin found that rock salts are a significant contributor to increased chloride levels in bodies of water and that these increased levels may persist for years due to chronic inputs of salt. Deicing salts increase the salinity of waters which can limit the growth of and even have lethal effects on organisms which live in these aquatic environments [17–19]. Additionally, increases in salinity due to rock salt contamination can result in changes to aquatic community structures and food webs as salt intolerant organisms can no longer survive [20]. Similarly, the leaching of rock salt compounds can make soils uninhabitable and toxic to some vegetation [21]. Another concern with rock salt accumulation in soil is that cations in the deicer may increase the rate at which heavy metals infiltrate groundwater which could threaten aquatic ecosystems and drinking water for humans [22]. In addition to heavy metal infiltration, increases in the salinity of drinking water caused by rock salt contamination could cause threats to humans who must follow low sodium diets for medical conditions such as high blood pressure or kidney disease [23]. Humans as well as their pets are also susceptible to skin and eye irritation if they come in contact with chlorides and other deicer chemicals [23, 24].

### Resident’s use and misapplication of fertilizers

A 2016 survey across 16 U.S. cities showed that 52 to 71% of people applied fertilizers to their lawns at least once a year [25]. These rates are consistent with [7, 8, 10, 26, 27], i.e. other research focused on fertilizer application to lawns, either by the homeowner directly or through hiring a contractor. While fertilization and lawn maintenance are common practices for American homeowners, many people may be misusing these materials. Fertilizer misapplication can occur two ways, either by the overapplication of fertilizer materials or by applying fertilizers to the areas that do not need them such as sidewalks and driveways [27]. In 1999, 54% of Chesapeake Bay homeowners reported using the instructions on the fertilizer bag as a guide for application while the next most popular option (13%) was for the homeowner to decide the amount to apply on their own [28]. This same survey found that only 16% of homeowners had received or conducted a soil test to determine the amount of fertilizer needed for their lawn [28]. These soil tests are crucial for determining the nutrient needs of a lawn to avoid over- or under-fertilizing [29]. In Cary, North Carolina, Osmond and Platt [30] found that 75% of the fertilizers being applied to lawns surpassed the industry standard limit of 20% nitrogen concentration. Cary residents also struggled with keeping their fertilizer applications off impervious surfaces or sweeping away excess fertilizers [30]. In Durham County, North Carolina 80% of respondents did not know how to calculate rates of fertilizer application for their lawns and only 20% understood that the three numbers on fertilizer labels [e.g., 10-10-10] represent percent nitrogen, phosphorus, and potassium [27]. Despite these challenges and gaps in knowledge amongst homeowners, a survey in Madison, Wisconsin, reported that an overwhelming majority (90%) of respondents acknowledged that lawn and residential fertilizer usage did have environmental implications, such as water quality issues in lakes, rivers, and ponds [31].

### Fertilizer usage

To American homeowners, the lawn is a reflection of the homeowner’s character as well as a sign of social commitment and economic investment [32]. A firmly established culture of maintaining an attractive landscape to achieve social acceptance and display commitment to one’s neighborhood exists in the United States [8]. Fraser et al. [10] and Fleming [27] found that residents in a neighborhood with a Homeowners Association (HOA) may be more likely to apply fertilizers to their lawn due to social pressure and regulations on well-kept lawns. The presence of an HOA was also associated with higher rates of fertilizer application, even though HOA representatives rarely intervene and community residents tend to self-regulate the maintenance of their lawns [10]. Oftentimes, homeowners with higher incomes are more likely to apply fertilizer chemicals to their lawns than lower income homeowners [7, 8, 10, 27, 28]. Other researchers [31] found that residents with higher incomes are more likely to identify agricultural fertilizers as a major pollutant to water resources, despite the trend of higher income individuals using fertilizers on their own lawns. There is some evidence that older landowners and those with higher levels of education are more likely to apply fertilizers to their lawn as well [7, 28]. However, research conducted across Nashville neighborhoods did not find higher levels of education to be correlated with higher uses of fertilizer [5, 6]. Additionally, higher education levels are associated with an increased willingness to seek advice on fertilizer practices and increased likelihood of implementing those suggested practices [28]. Women and rural landowners are less likely to apply fertilizers to their properties compared to men and suburban or urban landowners [8]. A homeowner’s decisions to use fertilizers is guided by social pressures, property values, and recreational use of their lawns for their children and pets [5, 6].

### Contractor and commercial uses and perceptions of deicers

Most of the research on deicer use focuses on commercial settings and the application of deicers to roads and highways. In 2021, highway deicing salt accounted for 42% of salt consumed in the United States [33]. Applying deicer salts to roads reduced car accidents by 88% and accident-related injuries by 85%, making this an important practice to allow drivers to safely access roads in inclement weather [34]. The amount of deicer being applied to roads and highways has continued to increase in recent years, especially with increases in urban land cover [35]. To mitigate and reduce water quality problems, municipalities may be incentivized to implement Best Management Practices (BMPs) to reduce deicer related pollution [36]. In commercial settings, contractors may be motivated to implement BMPs which involve reducing the amount of salt used per mile of roadway because this will reduce their overall expenses [36]. Winter maintenance officers from Wisconsin counties and major cities indicated that the most influential factor in choosing deicer types was effectiveness, precipitation and temperature in the weather forecast, and cost [37]. Environmental impact was rarely listed as a determinant of deicer selection [37].

### Residential and landowner uses and perceptions of deicers

While interest exists in the ways municipalities and contractors apply deicers to streets and highways, there are significant gaps in research focused on residential use, perceptions, and knowledge of deicer application. Surveys distributed to homeowners in two Wisconsin cities revealed that 22–26% of homeowners were unwilling to stop their use of deicer salts on their properties in the winter, however a majority of respondents (65% in 2013 and 72% in 2014) felt that reducing salt use would be effective or very effective in lessening water pollution [31, 38]. Those same survey respondents explained that some of the reasons they choose to apply deicers to their driveways and sidewalks are to prevent falls and injuries and to adhere to community mandates [31, 38]. There is a concern among deicer users that if they choose not to apply salt to their property they may contribute to injuries and be at risk of a lawsuit [31, 38]. Concern over human injuries and wellbeing may be given priority over addressing environmental issues, like water pollution [31, 38]. A survey conducted by the Michigan Department of Transportation found that residents did show concern regarding the environmental impacts of traditional deicing chemicals and were in favor of finding more environmentally friendly alternatives [39]. However, these residents were concerned that there are no affordable deicer alternatives and that the alternatives available may not be as effective [39]. Another common concern residents expressed about reducing deicer salts is that they were unfamiliar with what kind of alternatives exist [31, 38].

### Objectives and research questions

Exploring the gaps in homeowner understanding and use (or misuse) of fertilizer and deicer materials may be advantageous for designing more effective educational and outreach materials to change residents’ behavior and mitigate the negative environmental impacts of fertilization and deicing. The primary purpose of this research is to explore and compare residents’ understanding and application of fertilizer and deicer materials. To achieve this goal, two research questions were designed. First, how do residents in the study area, Hamilton County, Ohio, apply fertilizers and deicers to the properties at which they reside? This includes asking residents about the quantity of fertilizer and deicer being applied, the frequency with which residents use them, the methods people use to distribute these materials, and their general knowledge or reasoning to determine application amounts. Second, what factors influence residents’ decisions to use or not to use these materials on their property?

## Methods

### Study area

**Fig 1:**
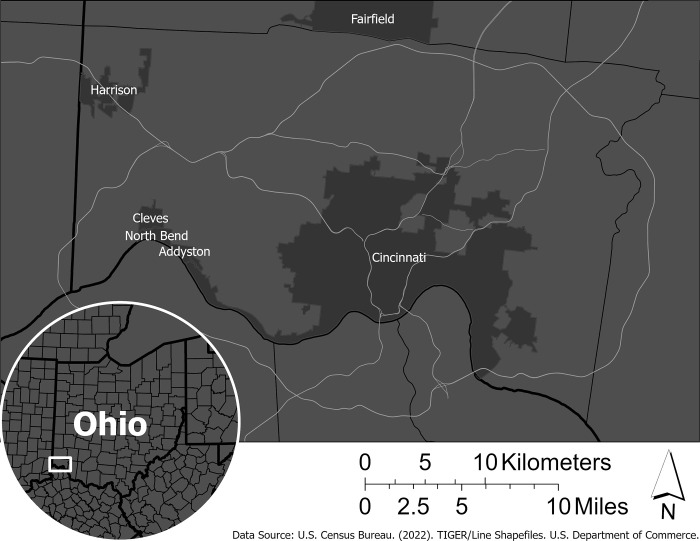
Study area map of Hamilton County, Ohio with inset for the State of Ohio. Major roads are shown for reference.

Hamilton County, Ohio was selected as the study area for this research (Fig 1). Located in Southwestern Ohio and within the Ohio River Valley, the county is one of multiple counties wherein sits the City of Cincinnati. The U.S. Census estimates that Hamilton County had a population of approximately 826,000 people as of 2021 [40]. Hamilton County is thought to be representative of a typical Midwestern county with a mixture of urban, residential, and rural areas and populations. We focused on Hamilton County (Ohio, USA) because its inhabitants (and their answers to our survey) will most likely represent the population in Midwestern USA, i.e. people who are prone to using fertilizers and deicers. Indeed, the Midwest is classified as a humid continental with hot or mild summers (depending on the state) and year-round precipitation; given the urbanized nature of the county (with driveways and sidewalks) as well as the ubiquitousness of residential turfgrass this area is ideally suited to study use and perceptions of fertilizers and deicers.

### Survey instrument

To address the aforementioned research questions, we designed a survey to gauge respondents’ perceptions and usage of fertilizers and deicers (S1 Fig). Questions included in the survey asked respondents about the frequency with which residents use fertilizer and deicer, perceptions and knowledge of these materials, and demographic information (e.g., age, income, education, gender). Previous studies which focused on individuals’ uses of fertilizers, deicers, and other lawn management practices [26–28, 31, 38, 39, 41] were used as a guide for designing questions for this survey.

We selected a random sample of 300 single family homes in Hamilton County to receive the survey materials using ArcGIS Pro 2.9.2 [42] and parcel data downloaded from the Hamilton County Community Planning Maps and GIS website in May of 2022. Previous surveys we conducted in this area with similar methods obtained response rates of 57% and 58% (Davis et al. [43] and Davis and Stoyko [44], respectively) but those surveys were extremely visual, topically appealing, and short so we expected lower response rates here. Our sampling population in this study is 236,623 single family homes within Hamilton County (Ohio, USA). To generalize the answers from the survey to the rest of the population at a 95% confidence level with a ±10% margin of error we needed 96 completed surveys [45]. By surveying 300 households, assuming a 40% response rate, we essentially ensured we met our minimum goal of 96 completed surveys. The surveys, as well as a $2 bill incentive, were distributed and collected using the Drop-off Pick-up (DOPU) method [45]. The dates of distribution and collection of survey materials took place from the first week of June through the third week of August 2022. Placing the fully or partially completed survey for the researchers to retrieve was understood as providing informed consent. The need for written consent was waived by the ethics committee. Completed survey responses were recorded using Qualtrics [46]. ArcGIS Pro 2.x was used to classify land cover and area for each household selected for surveying. The land covers on each parcel were digitized and divided into the following categories: lawn, building, driveway, sidewalk, patio, and pool. The various land cover classifications and their surface area for each parcel were used to calculate suggested fertilizers and deicer amounts for each household. These suggested amounts were compared to the amounts self-reported by respondents in the surveys.

### Statistical analyses

We performed all statistical analyses using R version 4.1.1 (2021-08-10). To examine whether there were differences in respondent’s opinions and perceptions of fertilizer and deicer use, we used paired t-tests to compare responses to fertilizer and deicer Likert-scale questions designed to mirror each other. We then performed one-way Analysis of Variances (ANOVAs) to compare demographic and socioeconomic characteristics to Likert-scale questions about fertilizer and deicer perceptions. Lastly, we performed multivariate regression analyses to ascertain whether socioeconomic and lifestyle characteristics of respondents could explain the variation in reported fertilizer use while controlling for lawn size.

## Results

The primary goal of this study was to compare the usage and perceptions of fertilizer and deicer among homeowners. We distributed surveys to a total of 283 households within Hamilton County, Ohio. In total, 110 surveys were returned for a response rate of 38.88%. Of the 300 households originally selected for surveying, 17 were unable to be surveyed (foreclosed, abandoned, or demolished) or the homeowner immediately declined to participate.

### Fertilizer usage

A total of 59 respondents (55.14%) reported using fertilizers on their lawns at least once a year (Table 1). Most fertilizer users report applying the materials from the months of March to October, however some do apply during winter months. Residents indicated that aesthetics, plant growth and health, and being neighborly were all motivators for applying fertilizers. However, when asked to identify the single most impactful determinant for choosing to apply fertilizers, 85.71% selected that they do so to maintain an attractive, green lawn. Also, over two-thirds (67.65%) of respondents who explained their methods for applying fertilizers explained that they rely on the instructions provided on the bag rather than their own judgement or by guessing (Table 1).

**Table 1:** Summary of respondents’ fertilizer usage.

Fertilizer Usage	n	Percent
Do you fertilize at least once per year?		
Yes^a^	59	55.14%
No	48	44.86%
Which months do you apply fertilizer?^b^		
January	1	0.56%
February	3	1.69%
March	29	16.29%
April	25	14.04%
May	21	11.80%
June	28	10.11%
July	14	7.87%
August	11	6.18%
September	24	13.48%
October	28	10.11%
November	13	7.30%
December	1	0.56%
How often do you apply fertilizer?		
Once a year	7	12.96%
Every few months	37	68.52%
Monthly	4	7.41%
More than once a month	1	1.85%
I’m not sure	5	9.26%
Have you ever tested your soil?		
Yes	9	14.52%
No	53	84.48%
If you have tested your soil, do you test yearly?		
Yes	3	27.27%
No	8	72.73%
Do you use granular or liquid fertilizer?		
Granular	33	62.26%
Liquid	9	16.98%
Both	11	20.75%
Why do you apply fertilizers?^b^		
To maintain my property’s appearance	52	46.85%
To support plant growth and health	39	35.14%
To meet HOA regulations	1	0.90%
It is the neighborly thing to do	13	11.71%
Other	9	5.41%
Do you sweep away excess fertilizer from impervious surfaces?		
Always	25	42.37%
Sometimes	12	20.34%
Never	9	15.25%
I am not sure	10	16.95%
Other	3	5.08%
Application Method		
Drop Spreader	14	20.90%
Broadcast Spreader	29	43.28%
Hand Spreader	6	8.96%
Spread By Hand	1	1.49%
I am not sure	7	10.45%
Other	10	14.93%
How do you determine how much to apply?		
Bag instructions	23	76.67%
Calculations based on lawn size	3	10.00%
Lawn care service decides	3	10.00%
Own judgement	1	3.33%

Questions ask respondents about frequency, methods, and motivations of fertilizer application.

^a^All following responses come from a subpopulation of those who answered “Yes” to the question “Do you fertilize at least once per year?”.

^b^Respondents were asked to select “All that apply”.

### Deicer usage

Most survey respondents do apply deicer to their properties (n = 73, 67.59%; Table 2). Months of deicer application only included those during which the Ohio county typically receives ice and snow accumulation (November to March). Deicer users are also most prompted to apply them following ice (45.04%) or snow accumulation (29.01%). Fewer users rely on forecasts for future ice or snow accumulation (12.98%) or freezing temperatures (8.40%). Out of all respondents who chose to answer an open-ended question to explain methods for deicer application 98% explained that they rely on their own judgement for the amount to apply. Only 1 person indicated that they refer to deicer package instructions (Table 2).

**Table 2:** Summary of respondents’ deicer usage.

Deicer Usage	n	Percent
Do you apply deicer at least once per year?		
Yes^a^	73	67.59%
No	35	32.41%
Which months do you apply deicer?^b^		
January	63	32.31%
February	64	32.82%
March	10	5.13%
November	10	5.13%
December	48	24.62%
What prompts you to apply deicer?^b^		
Weather forecasts predicting ice/snow	17	12.98%
Weather forecasts predicting freezing temperatures	11	8.40%
Snow Accumulation	38	29.01%
Ice Accumulation	59	45.04%
Other	6	4.58%
What kind(s) of deicer do you use?^b^		
Chloride-based	32	37.65%
Acetate-based	6	7.06%
Carbohydrate-based	1	1.18%
Non-salt “pet-friendly” options	21	24.71%
I am not sure	19	21.18%
Other	7	8.24%
How do you decide how much to apply?		
Bag instructions	1	2.00%
Own judgment	49	98.00%
Why do you apply deicers?^b^		
Safety reasons	65	30.23%
It is the neighborly thing to do	17	7.91%
To avoid liability from ice related injuries	26	12.09%
To help move vehicles to/from my driveway	38	17.67%
To make sidewalks easier to walk on	47	21.86%
To make my property more visually appealing	5	2.33%
To prevent ice related damages to my property	11	5.12%
Other	6	2.79%

Questions ask respondents about frequency, methods, and motivations of deicer application.

^a^All following responses come from a subpopulation of those who answered “Yes” to the question “Do you use deicer at least once per year?”.

^b^Respondents were asked to select “All that apply”.

### Fertilizer versus deicer perceptions

**Fig 2:**
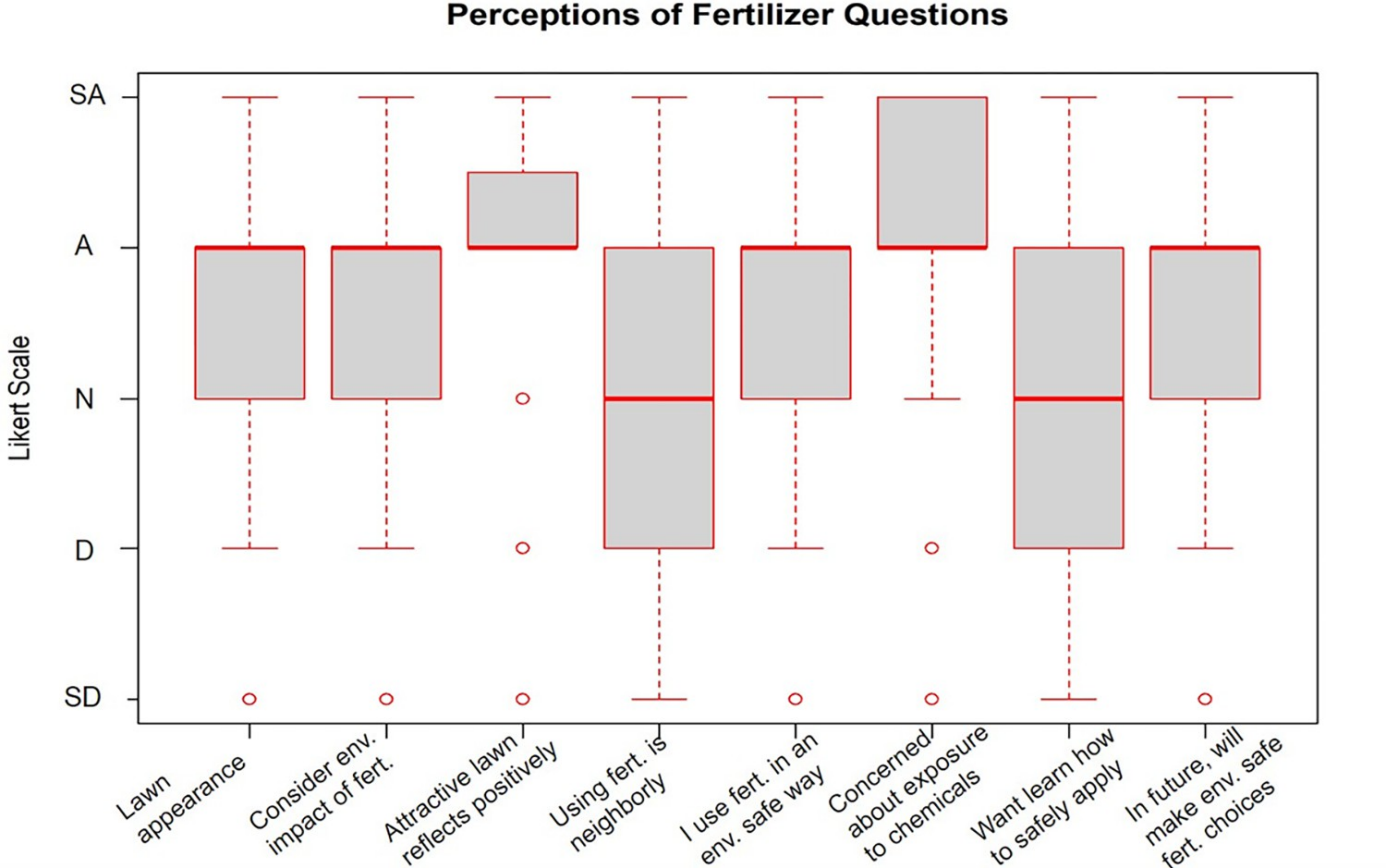
Boxplots of fertilizer perception Likert scale questions. SD stands for Strongly Disagree, A for Agree, N for Neutral, D for Disagree, and SD for Strongly Disagree. The full questions from left to right were: “It is important for me to have a lush green lawn”, “I take into consideration how my lawn care practices might affect the environment when applying fertilizer”, “Having an attractive lawn reflects positively on me as a resident in my neighborhood”, “Using fertilizers to have an attractive lawn is the neighborly thing to do”, “I use fertilizers in a way that minimizes negative environmental effects”, “I am concerned about myself, my family, or my pets being exposed to the chemicals in fertilizers”, “I am interested in learning more about how to properly and safely apply fertilizers to my property”, “In the future, I will consider making fertilizer choices which are safer for the environment”.

**Fig 3:**
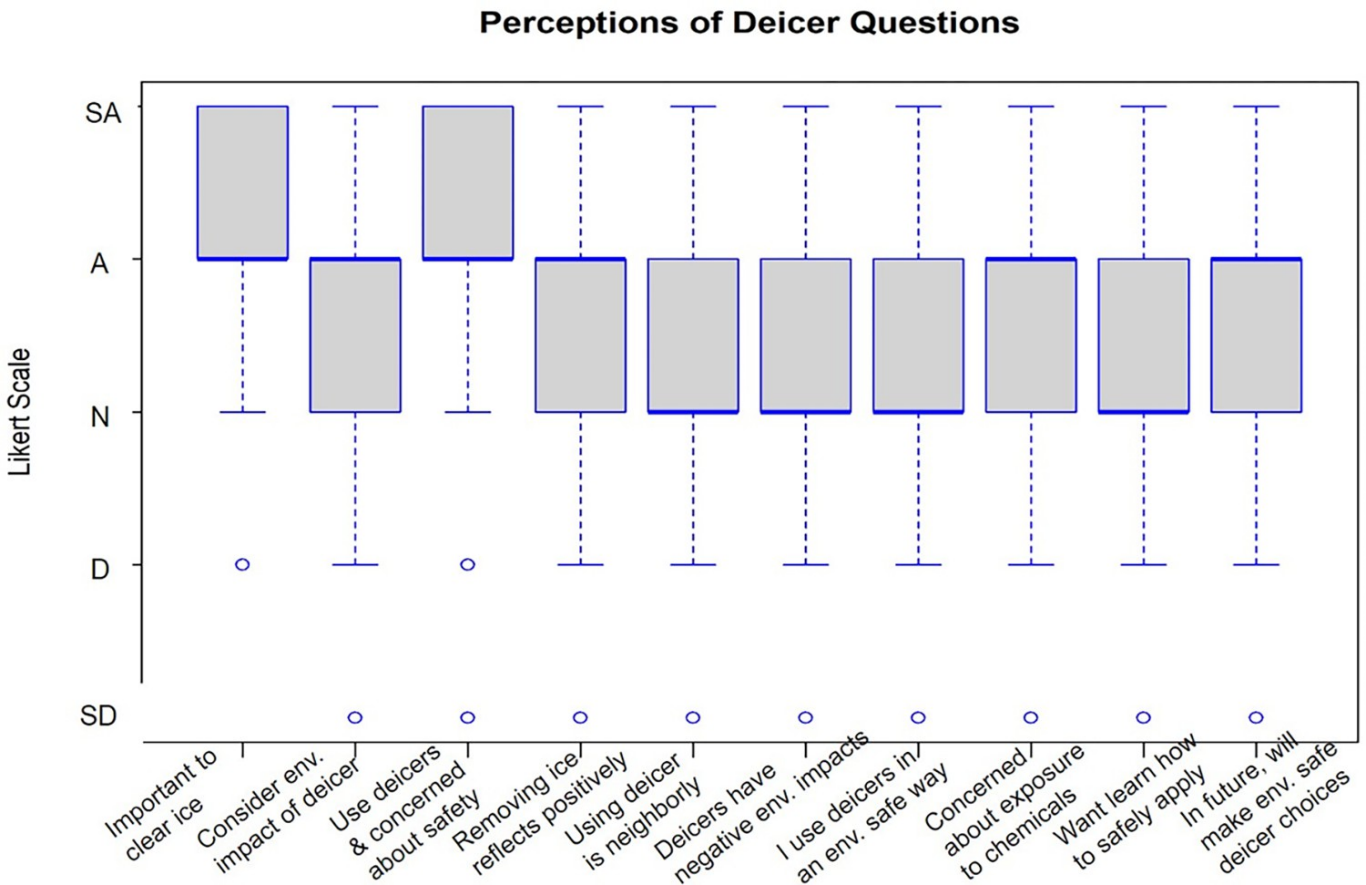
Boxplots of deicer perception Likert scale questions. SD stands for Strongly Disagree, A for Agree, N for Neutral, D for Disagree, and SD for Strongly Disagree. The full questions from left to right were: “It is important for me to remove an/ or prevent ice accumulation on my property”, “I take into consideration how my deicer use might affect the environment”, “I use deicers on my property because I am concerned about the safety of my family and myself”, “Removing ice from property reflects positively on me as a resident in my neighborhood”, “I use deicers on my property because it is the neighborly thing to do”, “Deicers contribute to negative environmental impacts”, “I use deicers in a way which reduces negative environmental impacts”, “I am concerned about myself, my family, or my pets being exposed to the chemicals in deicers”, “I am interested in learning more about how to properly and safely apply deicers to my property”, “In the future, I will consider making deicer choices which are safer for the environment”.

Perceptions of fertilizers and deicers were measured on a 5-point Likert scale with the options of strongly disagree (1), disagree (2), agree nor disagree (3), agree (4), strongly agree (5) (Figs 2 and 3). Means over three indicate more agreement with a statement whereas means under three indicate that overall, the respondents more strongly disagree with the statement. Tables 3 and 4 summarize these responses.

**Table 3:** Summary of fertilizer perception Likert scale questions.

Question	Mean +/- 1SD	n	Min/Max
It is important for me to have a green lawn	3.59 +/- 1.01	107	1–5
I consider how my fertilizer application affects the environment	3.66 +/- 1.04	95	1–5
Having an attractive lawn reflects positively on me among my neighbors	3.99 +/- 0.80	107	1–5
Using fertilizers to have an attractive lawn is a neighborly thing to do	2.96 +/1 1.06	103	1–5
I use fertilizers in a way that minimizes negative environmental impacts	3.73 +/- 0.94	79	1–5
I am concerned about myself, family, or pets being exposed to fertilizer chemicals	3.96 +/- 1.05	104	1–5
I am interested in learning more about how to properly and safely apply fertilizer	3.13 +/- 1.12	89	1–5
In the future, I will consider making fertilizer choices which are safer for the environment	3.67 +/- 0.97	92	1–5

Potential answers ranged from 1 to 5; 1 meaning strongly disagree, and 5 strongly agree. 1SD stands for one standard deviation from the mean, and n is the number of respondents who answered that question.

**Table 4:** Summary of deicer perception Likert questions.

Question	Mean +/- 1SD	N	Min/Max
It is important for me to remove / prevent ice accumulation on my property	4.07 +/- 0.74	100	2–5
I take into consideration how my deicer use affects the environment	3.47 +/- 1.00	85	1–5
I use deicers on my property because I am concerned about the safety of my family and myself	4.28 +/- 0.89	82	1–5
Removing ice from my property reflects positively on me among my neighbors	3.51 +/- 0.95	92	1–5
Using deicers on my property is a neighborly thing to do	3.25 +/- 1.00	77	1–5
Deicers contribute to negative environmental impacts	3.18 +/- 0.81	92	1–5
I use deicers in a way which reduces negative environmental impacts	3.41 +/- 0.87	81	1–5
I am concerned about myself, family, or pets being exposed to deicer chemicals	3.64 +/- 1.03	96	1–5
I am interested in learning more about how to properly and safely apply deicers	3.23 +/- 1.00	87	1–5
In the future, I will consider making deicer choices which are safer for the environment	3.62 +/- 0.89	87	1–5

Potential answers ranged from 1 to 5; 1 meaning strongly disagree, and 5 strongly agree. 1SD stands for one standard deviation from the mean, and n is the number of respondents who answered that question.

The Likert scale questions were designed to be able to compare residents’ perceptions of fertilizers versus their perceptions of deicers. There was significant evidence that residents more strongly agree that it is important for them to remove and or prevent ice accumulation from their property than it is for them to have a lush green lawn (t(96) = -4.872, p = 4.349e-06). There was evidence that homeowners were significantly more likely to take into consideration the environmental impact of their fertilizer use rather than the environmental impacts of deicer usage (t(73) = -2.162, p = 0.034). Respondents appeared to more strongly agree that using deicers to clear ice on their property is a more neighborly action compared to using fertilizers to maintain an attractive lawn (t(73) = -2.292, p = 0.025). Conversely, there was significant evidence that homeowners more strongly agreed that having an attractive lawn reflects more positively on them as a resident of their neighborhood than it does for them to remove ice from their property (t(89) = 4.747, p = 7.849e-06). Homeowners more strongly agreed that they use fertilizers in a way to reduce environmental impacts than they do when using deicers and the difference is significant (t(59) = 2.960, p = 0.004). There is evidence that homeowners were more concerned about their pets, children, and themselves being exposed to the chemicals in fertilizers than they are of being exposed to the chemicals in deicers (t(91) = 3.205, p = 0.002). There is no evidence that homeowners would rather learn more about properly/safely applying fertilizers vs deicers (t (77) = -0.815, p = 0.418). Also, there is no evidence that homeowners are more or less likely to consider making fertilizer or deicer choices which are safer for the environment (t(75) = 1.210, p = 0.230).

### Comparing self-reported fertilizer and deicer use to suggested use

Lawn sizes for surveyed parcels ranged from 910 to 226,998 ft^2^ (84 to 21,088 m^2^). Survey respondents were asked to report the quantity of fertilizer and deicer they apply to their property annually. The self-reported application amount was compared to a suggested application quantity calculated by using parcel areas for lawn fertilization and the combined area of driveways and walkways, for deicer application. A rate of 5 lbs. nitrogen per 1000 ft^2^ (2.268 kg per 92.9 m^2^) was used to calculate suggested fertilizer amounts [47]. Fertilizer application rates are based on the nitrogen content of the mixture [47]. Generally, lawn fertilizers have a nitrogen content of around 20%. For suggested deicer use, 0.6 lbs. per 1 ft^2^ (0.272 kg per 0.093m^2^) was the rate selected for calculations [48]. However, application rates should vary based on deicer material, severity of ice accumulation, and temperatures [49]. Only 29 respondents reported usable fertilizer application amounts. Of those 29, 14 (48.3%) reported using amounts that surpassed the suggested application rate. A total of 43 respondents reported the amounts of deicer applied annually. Fourteen of those 43 respondents (32.6%) reported using amounts of deicer which exceeded the recommended application amount for their total amount of impervious surface (other than roofs). Self-reported fertilizer and deicer amounts are summarized in Tables 5 and 6.

**Table 5:** Summary of self-reported fertilizer amounts applied by respondents.

	Mean +/- 1SD	Median	Min-Max
Fertilizer (lbs.)	67.19 +/- 68.55	40	5–252

Amounts were reported in pounds. (Amounts in kg: Mean +/- 1SD = 30.48 +/- 31.09; Median = 18.14; Min-Max = 2.27–114.32)

**Table 6:** Summary of self-reported deicer amounts applied by respondents.

	Mean +/- 1SD	Median	Min-Max
Deicer (lbs.)	28.84 +/- 31.16	20	0.5–150

Amounts were reported in pounds (lbs). (Amounts in kg: Mean +/- 1SD = 13.08 +/- 14.13; Median = 9.07; Min-Max = 0.23–68.04)

### Socioeconomic and lifestyle determinants of uses and perceptions of fertilizers and deicers

Respondents were asked to indicate various lifestyle and demographic characteristics (Table 7).

**Table 7:** Summary of socioeconomic and lifestyle characteristics of the survey respondents.

Socioeconomic and Lifestyle Variables	N	Percent	Mean	Min-Max
Belongs to an HOA				
Yes	16	14.81%		
No	91	84.26%		
I’m not sure	1	0.93%		
Pets play on property				
Yes	61	55.45%		
No	49	45.55%		
Children play on property				
Yes	43	39.09%		
No	67	60.91%		
Property Type				
Suburban	91	82.73%		
Urban	15	13.64%		
Rural	4	3.54%		
Employment Status				
Full-time	62	57.41%		
Part-time	1	0.93%		
Not Working	3	2.78%		
Retired	42	38.89%		
Student	0	0%		
Education				
High School	15	13.89%		
Certificate	2	1.85%		
Associate’s Degree	14	12.96%		
Some College	15	13.89%		
4 Year College Degree	35	32.41%		
Master’s Degree	21	19.44%		
Doctoral Degree	6	5.56%		
Age (Years)			59.38	28–94
Gender				
Male	63	57.80%		
Female	46	42.20%		
Race and Ethnicity				
White	98	91.59%		
Hispanic	1	0.93%		
African American	7	6.54%		
American Indian	0	0%		
Asian/ Pacific Islander	0	0%		
Other	1	0.93%		
Household Income				
<$25,000	4	4.76%		
$25,000 up to $50,000	18	21.43%		
$50,000 up to $100,000	17	20.24%		
$100,000 up to $150,000	29	34.52%		
More than $150,000	16	19.05%		

An ANOVA was conducted to determine if household income explains the degree to which a respondent feels that having a green, lush lawn is important to them. Income was selected as a variable on which to focus based on a literature review and strong correlation between it and the left-hand side variable (results not shown). A one-way ANOVA revealed that there was a statistically significant difference between the mean perception of having a green, attractive lawn based on income level (Table 8).

**Table 8:** Summary of perceptions of having a green lawn based on household income and Tukey HSD comparison results (F (4,77) = 4.102, p = 0.005).

Income Level	Mean +/- 1 SD	Median	n	Min-Max	Tukey HSD Comparison				
					$0–25,000	$25,000–50,000	$50,000-$100,000	$100,000–150,000	>$150,000
$0–25,000	2.75 +/- 0.5	3	4	2–3					
$25,000–50,000	3.059 +/- 0.966	3	17	1–4	0.977				
$50,000-$100,000	3.647 +/- 1.115	4	17	2–5	0.446	0.384			
$100,000–150,000	3.724 +/- 0.956	4	29	2–5	0.320	0.163	0.999		
>$150,000	4.266 +/- 0.799	4	15	2–5	0.047*	0.005*	0.364	0.390	

Respondents were asked how strongly they agree with the statement “It is important for me to have a lush, green lawn”. 1SD stands for one standard deviation from the mean, and n is the number of respondents who answered that question.

Two sample t-tests were conducted to determine if differences exist between pet owners and non-pet owners regarding concern over these materials. Respondents who own pets are significantly more concerned over their pet(s) exposure to the chemicals in fertilizers (Table 9). This is true for deicers as well (Table 9).

**Table 9:** Independent sample t-test results examining concern for exposure to fertilizer and deicer materials between respondents with and without pets.

Pets	No Pets			
Mean +/- 1SD	Mean +/- 1SD	d.f.	t	p
4.150 +/- 1.006	3.705 +/- 1.069	102	2.173	0.032*
3.887 +/- 1.013	3.326 +/- 0.993	94	2.723	0.008*

The first line represents the statement, “I am concerned about my family or pets being exposed to the chemicals in fertilizer”. The second line represents the statement, “I am concerned about my family or pets being exposed to the chemicals in deicer”. 1SD stands for one standard deviation from the mean, and n is the number of respondents who answered that question.

Two sample t-tests were also conducted to determine if there exist differences between those with and without children and their concern about exposure to fertilizer or deicer chemicals. There was no statistically significant difference between homeowners with or without children and their concern about exposure to chemicals in fertilizers or deicers (Table 10).

**Table 10:** Independent sample t-test results examining concern for exposure to fertilizer and deicer materials between respondents with and without children.

Children	No Children			
Mean +/- 1SD	Mean +/- 1SD	d.f.	t	p
4.167 +/-1.010	3.823 +/- 1.064	102	1.651	0.102
3.651 +/- 1.115	3.649 +/-0.991	94	-0.156	0.877

The first line represents the statement, “I am concerned about my family or pets being exposed to the chemicals in fertilizer”. The second line represents the statement, “I am concerned about my family or pets being exposed to the chemicals in deicer”. 1SD stands for one standard deviation from the mean, and n is the number of respondents who answered that question.

A simple linear regression was performed to examine the relationship between age and respondents’ perceptions of the importance of clearing or preventing ice accumulation from their properties. There was no evidence of a relationship between these two variables; F(1,96) = 0.3238; p = 0.571, r-squared = -0.007. Additionally, multivariate linear regressions were used to explain the amount of fertilizer used with the following predictors: lawn size, income, age, education, employment status, and property type (see Appendix C for regression diagnostic plots). The best model included income and age as predictor variables. This two-predictor model explains 39.76% of the variance in the amount of fertilizer used by each respondent; F(5,17) = 3.904; p = 0.0154 (Table 11).

**Table 11:** Summary of stepwise regression model to explain reported amounts of fertilizer used (adjusted r-squared = 0.398).

Predictor	Estimate	SE	95% CI		p
			LL	UL	
Intercept	-81.818	58.199	-199.962	32.922	0.149
Income ($25,000-$50,000)	1.653	0.0003	-94.521	85.317	0.915
Income ($50,000-$100,000)	-6.653	46.192	-34.483	138.389	0.222
Income ($100,000-$150,000)	51.312	42.463	-35.516	130,589	0.244
Income (>$150,000)	47.055	40.705	47.269	229.981	0.005**
Age	138.083	44.782	0.172	3.195	0.031*

## Discussion

### Fertilizer usage among residents

According to the survey results, slightly more than half of homeowners in our sample report use fertilizers on their lawns at least once per year. This is consistent with [25] which reported that fertilizer usage rates tend to be between 52 to 71%. Fleming [27] has identified significant gaps in homeowner understanding of how to properly apply fertilizers to their lawns. Additionally, a survey from Swann [28] found that only half of homeowners refer to bag instructions when deciding how much fertilizer to apply to their lawns. However, most respondents (76.67%) in this study reported being comfortable using the manufacturer’s instructions on the packaging as a guide for use and application [25]. Despite most of the respondents stating that they reference the recommended directions, our analysis revealed that a nearly half (48%) of respondents reported using annual quantities of fertilizer which exceeded the recommended amounts for their lawn size on average by 58.44 lbs (26.51 kg) annually.

There are multiple factors which may have contributed to the application of excessive fertilizer. Residents might be miscalculating the appropriate amount of fertilizer to account for the nutrients that are or are not present in their soil. Testing the pH of soil can accurately determine the nutrients present in a lawn which could help residents identify if fertilizer use would be beneficial for treating any deficiencies [29, 50]. The nutrient profiles of soil in urban and residential areas can vary significantly based on different regional and environmental factors which is why homeowners should test their soil before applying fertilizer treatments [51]. In addition to using soil tests or even visual inspections of the state of the lawn’s health, people should also consider the area of lawn being treated in order to calculate the proper amounts of fertilizer needed [52]. Additionally, those applying fertilizers to their property need to be aware of the type of fertilizer they are applying as fast-release, slow-release, and organic fertilizers have different N-P-K ratios [52]. However, this survey and previous studies [28, 29, 38] found that an overwhelming majority of homeowners do not attempt to test their lawns for pH and nutrient levels.

The frequency with which homeowners apply fertilizer to their lawns can also contribute to the accumulation of excess fertilizer. Most respondents who use fertilizer reported treating their properties with multiple applications throughout the year. Many fertilizer manufacturer instructions do encourage reapplication every few months, however, too many applications or applying fertilizer at the wrong times may be ineffective or contribute to negative environmental impacts. The U.S. Environmental Protection Agency [53] reports that spring and summer fertilizer applications are the most effective for warm-season grasses, while cool-season grasses experience peak nutrient uptake during the summer and fall. While most respondents here reported applying fertilizers during spring, summer, and early fall, there were multiple people (7%) who selected November as a time for fertilizer application. Some (3%) even reported applying during the winter months of December, January, and February. Fertilizer applications during late fall and winter seasons result in reduced nutrient uptake making it more likely that excess nutrients will runoff into surrounding water sources [54].

Another issue which may contribute to excessive fertilizer use is how homeowners decide to apply the materials. Various methods that can be used to distribute fertilizer include broadcast spreaders, drop spreaders, hand spreaders, or spreading by hand. Hand spreaders tend to provide the most accurate and even distribution of granular fertilizer, whereas broadcast spreaders more often result in excessive application amounts [55]. A majority of respondents in SW Ohio favored the broadcast spreader over other methods which may explain why so many people appear to be using inappropriate amounts of fertilizer for their lawn size.

In addition to excessive application amounts, Fleming [27] and Osmond and Platt [30] have identified that another common environmental issue among homeowners who use fertilizers is that they often fail to sweep away what is spread onto impervious surfaces, like driveways and sidewalks. Sweeping away excess fertilizers is a BMP which prevents the spread of these materials into unintended areas, including waterways [27]. Of those surveyed in Hamilton County, a considerable number of respondents (32%) either do not sweep away fertilizer or are unsure if that is done at their property. This kind of behavior paired with the application of excess amounts of fertilizer may contribute to negative environmental impacts if these materials are washed or blown away into nearby sources of water and other areas which were not intended to have fertilizer treatments [27].

### Motivations behind fertilizer usage

The primary motivator for using fertilizers among respondents was to enhance the appearance of their turfgrass. Blaine et al. [8] and Carrico et al. [6] found that American homeowners tend to place great importance on maintaining an attractive, green lawn. While there might be individual preferences that motivate homeowners to fertilize their lawn, some suggest that the decision to use fertilizer to achieve a “beautiful” lawn is the result of social norms and the expectation to fit in with your community [5]. Past research has explained that there are outside influences on homeowner decision making which pressures them to maintain a manicured lawn in order to promote cohesion in their neighborhood and to show respect for their neighbors [5, 7]. Survey respondents in Oregon felt that their neighbors would dislike them if they failed to maintain a beautiful property [56]. This pressure motivated them to utilize lawn care practices including fertilizer application, regular watering, and the use of herbicides [56]. The respondents of this survey strongly agreed that using fertilizer to maintain their lawn reflects positively on them in their neighborhood which would support this idea that there might be complex social pressures which motivate homeowners to use fertilizer. Here, 13 respondents (11.71%) explained that they choose to use fertilizers because they think of it as a neighborly action. While this only represents a small percentage of the respondents, these answers still suggest that at least some portions of people are choosing to make their lawn care choices based on the desire to be accepted by their neighbors and do so consciously. While this study asked individuals to focus on their own uses and perceptions, a questionnaire distributed to Ohio homeowners asked respondents to consider their neighbors’ practices. That survey found that people did think that their neighbors’ use of lawn chemicals (e.g., fertilizers, herbicides) reflected positively on community pride and contributed to increased property values [8]. So, the concern of individuals to be accepted by their neighbors may be rooted in actual social expectations.

While complex social pressures informally shape the landscape choices of homeowners, organizations, such as HOAs, can enforce requirements and more strictly regulate the use of fertilizers (or inadvertently drive their overuse) to encourage cohesive landscape aesthetics throughout the community. HOAs may not require the use of fertilizers but might have written rules and expectations for households to maintain manicured, green lawns. Cook et al. [57] and Fraser et al. [10] report that people who belonged to HOAs and similar organizations were more likely to use fertilizers than those who did not. In this study, 15.74% of respondents belonged to an HOA but only one person stated that their fertilizer use was a result of pressure to adhere to HOA rules and regulations.

In addition to aesthetics and perceived or real social acceptance, the second most prominent motivator among these respondents was to use fertilizers to improve the health and growth of their lawns. These fertilizer users might still be swayed by individual preferences and social pressures; however, they are more concerned with maintaining a landscape in a way that improves the quality of their lawns and gardens. When applied in safe amounts, fertilizers can improve the health of a lawn by strengthening root systems and increasing nutrient up-take [58]. However, there are other best management practices (BMPs), such as using nonchemical organic fertilizers, which result in healthier lawns compared to practices that include the use of traditional chemical fertilizers [59]. For the respondents who identify lawn and plant health as their motivator for fertilizer usage, their behavior and willingness to change to various lawn BMPs could be shaped by proper education and outreach focused on explaining the benefits and successes of using organic and non-chemical-based methods for lawn maintenance [2].

### Deicer usage among residents

More than two-thirds of survey respondents (67.59%) reported using deicer on their properties at least once per year. Limited studies analyzing the usage rates of deicers on private residences exist, however, Sleeper [60] identified that the Northeastern and Midwestern states, including Ohio, account for the majority of winter rock salt purchases (75%). Hamilton County, Ohio, is located within the Midwest and experiences varying levels of winter weather severity each year [61]. Annual differences in snow and ice accumulation may impact the rates and regularity of deicer application used by households. In this study, respondents reported using deicer materials between the months of November and March; though, December, January, and February tend to receive the most deicer treatments, likely because ice and snow accumulation is more consistent during these months.

Homeowners are primarily prompted to apply deicers to their properties after winter weather has already occurred. Ice accumulation was the first deciding factor for people to apply deicers (45.04%) while snow accumulation was the second most important (29.01%). Deicers are typically used in response to winter weather instead of being used as a preventative measure. While most apply deicers after ice and snow has formed, using anti-icing agents, such as liquid salt brines, before winter weather starts can prevent ice from forming and ultimately reduce the quantity of salt people would need to apply to their surfaces [62, 63]. This too could be disseminated via outreach materials and programs.

While it is possible to quantify suggested amounts of fertilizers people should apply to their lawns and gardens based on surface area, determining the amount of deicer to apply is considerably more challenging. Deicer packaging may suggest rates for application, but the type of deicer, surface area, temperature, ground temperature, and weather conditions can all play a part in determining how much deicer is necessary [49]. Chloride-based (salt) deicers represented 37.65% of the materials used by survey respondents while the second most popular type was non-chloride pet safe deicers (24.71%). Other non-salt alternatives which are not specifically marketed for protecting pets, including beet juice, do not appear to be popular among respondents in SW Ohio. Most respondents explained that they decide how much deicer to apply based on the extent of ice and snow cover on their properties rather than referring to the manufacturer recommendations. Because residents rarely utilize the instructions for their deicer materials, one of the most important BMPs they can utilize is to perform regular performance evaluations of their deicers after each application and storm event [62]. Taking note of what application rates and placements work and responding to different storm conditions can reduce overall salt application by up to 50% [62].

Impervious surfaces including driveways, sidewalks, and front steps and porches receive the most deicer applications among our respondents. Patio spaces are much less likely to receive any deicer treatment. The majority of respondents try to direct their applications only to the parts of the driveway, sidewalk, and front steps with the most ice or snow accumulation, rather than applying deicers across the entire areas. By focusing their application only to specific areas applying the material evenly to those portions of the surfaces, people will be able to reduce the total amount of deicer used.

### Motivations behind deicer usage

To the best of our knowledge, the reasoning behind deicer usage has been largely unexplored in previous research and one of the overarching goals of this study was to explore these motivations and pressures. Unlike fertilizer usage which is primarily motivated by aesthetic preferences and social pressure, people choose to use deicing materials as a safety measure against injuries caused from falling on ice patches. While residential uses of deicers have not been studied in depth, it is well established that the commercial application of deicers to roads and highways makes roadways safer and more accessible following periods of ice accumulation [63]. Along those same lines, residents are also applying deicers to increase the ease of moving their vehicles in and out of their driveways (17.67%) and to improve the accessibility and mobility of walking on sidewalks and steps (21.86%). Based on the lack of respondents who identified using alternative techniques or non-salt deicing materials, the application of deicer salts to remove ice and snow appears to be the only widely known option for making pathways and driveways safely accessible during winter weather [31, 38]. Consequently, respondents may feel that the use of deicers on their properties is a necessity as it is the only effective option to guarantee the safety and mobility of their household members and visitors. In contrast, the choices to use fertilizers come from complex social pressures and personal preferences to maintain an attractive property and contribute to a sense of community and social cohesion. Understanding these key differences may prove to be useful for education and outreach programs attempting to correct management practices which contribute to the degradation of ecosystems and water resources.

### Perceptions of fertilizer and deicer materials

In addition to examining the usage of deicers and the motivations of homeowners to use deicers, this research was aimed at comparing those aspects against those of fertilizer. Robbins et al. [7] explains that the idea of maintaining an attractive, green lawn is quite popular among American households. and the respondents of this survey strongly agree that there is value in using practices that contribute to a beautiful landscape. Respondents also strongly believe that it is important to maintain their properties during periods of winter weather by removing ice and snow accumulation from driveways and walkways. When these two perceptions are compared, removing ice and snow from a property is a significantly more important action than maintaining an attractive green lawn.

Respondents in this study do try to consider the impact that fertilizer has on the environment. There appears to be significantly less consideration for the environmental impacts of deicer use. Along these same lines, even though respondents report trying to use both materials in an environmentally friendly way, they are significantly more focused on applying fertilizers in a way that will reduce the impacts on the environment compared to deicers. Also, respondents only slightly agree that deicers contribute to negative impacts on the environment. Our findings may suggest that there are knowledge gaps among the public and their understanding of how deicers impact the environment. The issues of eutrophication from excess nutrients and freshwater salinization from salt runoff following rock salt application are both issues that have been present in the United States for decades [64, 65]. Despite this fact, people might be more familiar with the environmental impacts associated with fertilizer use than they are with the impacts of deicer. Ohioans specifically are likely familiar with the eutrophication problems of Lake Erie which is at the northernmost part of the state. Lake Erie consistently deals with harmful algal blooms (HAB) and dangerous cyanobacteria which contributes to the closing of beaches and other access points during peak summer seasons and is regularly reported on [66, 67] but no similar reports of environmental impacts of deicer use seem to be reported by the news, and do not seem to affect recreation in noticeable ways from the perspective of the public.

A compounding issue might be that for those who are aware of the environmental impacts of deicers, they might prioritize the use of deicers for safety reasons over the importance of aesthetics or reducing environmental degradation. Kollmuss and Agyeman [68] postulate that most people are going to prioritize the wellbeing of themselves and their family so when a pro-environmental behavior goes against this personal priority, people are less likely to adopt that behavior. Additionally, Liu et al. [69] analyzed data from the 2010 Chinese General Social Survey and found that knowledge of environmental degradation might not actually be enough to encourage pro environmental behavior and BMPs. Instead, environmentally protective practices might only be adopted by people after developing positive emotions and attitudes towards the environment [69]. Therefore, in regard to deicer usage, where homeowners have strong attitudes towards the safety of their families, residents might only be motivated to utilize deicer BMPs if they develop equally strong attitudes towards the environmental impacts of deicers. This will be key to developing associated outreach and educational materials and programs.

Focusing on the social perceptions of fertilizer and deicer usage, respondents thought that using fertilizers to achieve an attractive lawn reflects significantly more positively on them than it does for them to use deicers to clear ice accumulation. In contrast, when asked to select how neighborly both activities are, respondents felt that using deicers on their properties is a significantly more neighborly action than using fertilizers. Most were ambivalent towards the idea that using fertilizers to have an attractive lawn would be considered neighborly at all (or did not admit it or were not conscious of it). The thought that fertilizer usage and having a green lawn reflects more positively on a resident in their neighborhood aligns with the idea that there are complex social pressures which make people wish to be perceived more positively within their community [5]. This idea revolves more around a person’s social status and how much their neighbors are willing to embrace them as an accepted member of the group. However, when respondents report feeling that using deicers to clear ice off their property is more neighborly, this is referring to how that action would be thought of as considerate and supportive towards the other members of the neighborhood. While there are slight differences between the two concepts, both are rooted in the idea of being valued, respected, and included within the community. Nassauer et al. [70] found that cultural and local norms considerably impact the degree to which residents maintain their yards and properties.

Survey respondents stated they were not interested in learning more about the environmental impacts of fertilizers. Most already declared they consider the environmental impact of fertilizers and try to use them in a way that will not contribute to harmful environmental effects, therefore people might feel that they already have enough knowledge on the topic. Respondents did express a slight interest in learning more about the environmental impacts of deicers and agreed that in the future they would try to use both deicers and fertilizers in ways that will reduce negative environmental impacts. However, residents actually choosing to implement more environmentally friendly lawn and property practices is complicated and Carrico et al. [6] report that this depends on a combination of social pressures, environmental concerns, and personal preference. Robbins and Sharp [9] found that ultimately, social obligations and the pressures to adhere to community expectations have more influence on homeowner behavior than their concerns for the environment. This is exemplified by the fact our respondents used more fertilizer than suggested by the manufacturer for their lawn size.

### Socioeconomic and lifestyle relationships with fertilizer and deicer usage

Higher amounts of applied fertilizer were mostly accounted for by the lifestyle and demographic characteristics of income and age. Higher incomes (>$150,000) were associated with higher amounts of fertilizer on lawns. In this survey, respondents with higher incomes more significantly valued the traditional American lawn with manicured, lush green grass. Therefore, they may be more liberally applying fertilizers to enhance the appearance of their yards. Robbins [71] also found that a homeowner is more likely to intensely fertilize their yard if they are older, have a higher income, and live in the Midwest or the South. However, Robbins [71] also identified higher education as an influence on fertilizer use, which this research did not find. Conversely, Fraser et al. [10], found that homeowners of higher and lower incomes judge lawn care as important but higher income households have more income available to purchase and apply fertilizer and they also view this lawn care action as a worthwhile return on investment due to the perceived impacts of manicured turf on home value. Fraser et al. [10] and Law et al. [72] also found that larger lots, which were correlated with higher incomes and house values, typically applied fertilizers at lower rates.

Those who do own pets seem to be concerned about the risks of their pets and their families being exposed to the chemicals in both deicer and fertilizers. However, even though concern surrounding exposure to chemicals exists, homeowners still purchase and apply fertilizers, pesticides, and other potentially harmful materials to their properties [71]. While concerned homeowners might still utilize fertilizers and other chemicals to maintain their properties, Campbell et al. [73] found that those with pets and children are more likely to consider purchasing organic or pet-/kid-friendly products. Based on the survey responses, there are a considerable number of homeowners using non-salt deicing products marketed as pet safe and this appears to be the most popular alternative to traditional salting. The respondents who use pet-friendly materials are still deicing to improve the safety of their walkways and driveways, yet they also want to take the safety of their pets into consideration and reduce exposure to harmful, irritating chemicals.

The survey responses regarding the value of using deicers to clear ice and snow accumulation from properties did not illustrate any significant relationship between age and how important people perceive this practice to be. However, a study by Wennberg et al. [74] exploring mobility of older people within their communities highlighted the concern among older people for having walkways clear of ice and snow. In a survey conducted among community-dwelling people aged 75 to 90, 53% of respondents identified ice and snow cover as a common barrier to their ability to safely navigate their communities [75]. Additionally, women within older age groups typically showed stronger concern about mobility on slippery, icy surfaces compared to men of the same age [74]. This research did not identify any significant relationship between age or gender regarding respondents’ perception on the importance of clearing ice and snow. These findings align with Li et al.’s [76] results from a Toronto based survey which also found no significant differences between age and the concern for icy surfaces. People of all ages and those with or without functional limitations showed equal concern for mobility on sidewalks during icy winter weather conditions [76].

## Conclusion

While applying fertilizers and deicers are both common practices among residents in the Midwest, we find that there are notable differences in the perceptions and uses of these materials. Fertilizer is primarily used by residents to achieve specific lawn aesthetics which helps them comply with complicated social pressures of maintaining an attractive, manicured lawn. Those with higher incomes were more likely to apply more fertilizers. In contrast, the primary motivation for applying deicers to sidewalks and driveways stems from the goal of creating safe, walkable pathways for the residents in a household. Neither income nor age of residents predicted how much they are concerned about clearing ice from sidewalks and driveways.

When applying fertilizers, residents stated that they typically refer to the instructions listed on the bag to guide the amount and frequency of use. In contrast, 98% of deicer users decide the amount and frequency of use based on their own judgment and the severity of the weather and most do “spot” applications, i.e. uneven applications whereby they apply more of the materials where there snow/ice has accumulated. Despite residents utilizing fertilizer bag instructions, most still overfertilize relative to their lawn size.

Overall concern with exposure to chemicals from fertilizers and deicers is moderately high, especially for pet owners. Additionally, residents were more likely to acknowledge the negative environmental impacts from the improper usage of fertilizers compared to deicers. It seems that people are much more concerned with maintaining safe, walkable surfaces than engaging in pro-environmental behaviors when considering deicer applications. Developing outreach materials and educational programs focused on strategies for deicer BMP adoption, such as pre-treating surfaces before ice accumulation, and the associated benefits (especially in terms of clean water for recreational purposes, but also addressing chemical exposure concerns for pets and children) could improve the environmental and social outcomes of residential deicer application.

## Supporting information

S1 Fig Survey instrument. (PDF)

S2 Fig Diagnostic plots for multivariate regression explaining deviations from suggested fertilizer amounts with income, age, and education. (TIF)

## References

[1] Clement DL, Malinoski MK. Homeowner Landscape Series: Common Cultural and Environmental Problems in Landscapes. 2011. doi: 10.13016/0PWT-KGWJ

[2] Suh DH, Khachatryan H, Guan Z. Why do we adopt environmentally friendly lawn care? Evidence from do-it-yourself consumers. Appl Econ. 2016;48: 2550–2561. doi: 10.1080/00036846.2015.1125431

[3] Levelton Consultants, Levelton Consultants Limited. Guidelines for the selection of snow and ice control materials to mitigate environmental impacts. Transp Res Board. 2007;577.

[4] Savci S. Investigation of Effect of Chemical Fertilizers on Environment. APCBEE Procedia. 2012;1: 287–292. doi: 10.1016/j.apcbee.2012.03.047

[5] Carrico AR, Fraser J, Bazuin JT. Green With Envy: Psychological and Social Predictors of Lawn Fertilizer Application. Environ Behav. 2013;45: 427–454. doi: 10.1177/0013916511434637

[6] Carrico AR, Raja US, Fraser J, Vandenbergh MP. Household and block level influences on residential fertilizer use. Landsc Urban Plan. 2018;178: 60–68. doi: 10.1016/j.landurbplan.2018.05.008

[7] Robbins P, Polderman A, Birkenholtz T. Lawns and Toxins. Cities. 2001;18: 369–380. doi: 10.1016/S0264-2751(01)00029-4

[8] Blaine TW, Clayton S, Robbins P, Grewal PS. Homeowner Attitudes and Practices Towards Residential Landscape Management in Ohio, USA. Environ Manage. 2012;50: 257–271. doi: 10.1007/s00267-012-9874-x 22638651

[9] Robbins P, Sharp J. Producing and Consuming Chemicals: The Moral Economy of the American Lawn. Econ Geogr. 2015;79: 425–451.

[10] Fraser JC, Bazuin JT, Band LE, Grove JM. Covenants, cohesion, and community: The effects of neighborhood governance on lawn fertilization. Landsc Urban Plan. 2013;115: 30–38. doi: 10.1016/j.landurbplan.2013.02.013

[11] Gerbino-Bevins BM, Tuan CY, Mattison M. Evaluation of Ice-Melting Capacities of Deicing Chemicals. J Test Eval. 2012;40: 104460. doi: 10.1520/JTE104460

[12] Ramakrishna DM, Viraraghavan T. Environmental Impact of Chemical Deicers–A Review. Water Air Soil Pollut. 2005;166: 49–63. doi: 10.1007/s11270-005-8265-9

[13] Stefan H, Novotny E, Sander A, Mohseni O. Study of Environmental Effects of De-Icing Salt on Water Quality in the Twin Cities Metropolitan Area, Minnesota. St. Anthony Falls Laboratory. Univ Minn Digit Conserv. Available: https://hdl.handle.net/11299/132320

[14] Li Y, Fang Y, Seeley N, Jungwirth S, Jackson E, Shi X. Corrosion by Chloride Deicers on Highway Maintenance Equipment: Renewed Perspective and Laboratory Investigation. Transp Res Rec J Transp Res Board. 2013;2361: 106–113. doi: 10.3141/2361-13

[15] Struzeski E. Environmental Impact of Highway Deicing. 1971.

[16] Godwin KS, Hafner SD, Buff MF. Long-term trends in sodium and chloride in the Mohawk River, New York: the effect of fifty years of road-salt application. Environ Pollut. 2003;124: 273–281. doi: 10.1016/s0269-7491(02)00481-5 12713927

[17] Corsi SR, Graczyk DJ, Geis SW, Booth NL, Richards KD. A Fresh Look at Road Salt: Aquatic Toxicity and Water-Quality Impacts on Local, Regional, and National Scales. Environ Sci Technol. 2010;44: 7376–7382. doi: 10.1021/es101333u 20806974 PMC2947309

[18] Herbert ER, Boon P, Burgin AJ, Neubauer SC, Franklin RB, Ardón M, et al. A global perspective on wetland salinization: ecological consequences of a growing threat to freshwater wetlands. Ecosphere. 2015;6: art206. doi: 10.1890/ES14-00534.1

[19] Hintz WD, Relyea RA. Impacts of road deicing salts on the early-life growth and development of a stream salmonid: Salt type matters. Environ Pollut. 2017;223: 409–415. doi: 10.1016/j.envpol.2017.01.040 28131472

[20] Collins SJ, Russell RW. Toxicity of road salt to Nova Scotia amphibians. Environ Pollut. 2009;157: 320–324. doi: 10.1016/j.envpol.2008.06.032 18684543

[21] Cunningham MA, Snyder E, Yonkin D, Ross M, Elsen T. Accumulation of deicing salts in soils in an urban environment. Urban Ecosyst. 2008;11: 17–31. doi: 10.1007/s11252-007-0031-x

[22] Bäckström M, Karlsson S, Bäckman L, Folkeson L, Lind B. Mobilisation of heavy metals by deicing salts in a roadside environment. Water Res. 2004;38: 720–732. doi: 10.1016/j.watres.2003.11.006 14723942

[23] Honarvar Nazari M, Mousavi SZ, Potapova A, McIntyre J, Shi X. Toxicological impacts of roadway deicers on aquatic resources and human health: A review. Water Environ Res. 2021;93: 1855–1881. doi: 10.1002/wer.1581 33978278

[24] Fay L, Shi X. Environmental Impacts of Chemicals for Snow and Ice Control: State of the Knowledge. Water Air Soil Pollut. 2012;223: 2751–2770. doi: 10.1007/s11270-011-1064-6

[25] Groffman PM, Grove JM, Polsky C, Bettez ND, Morse JL, Cavender-Bares J, et al. Satisfaction, water and fertilizer use in the American residential macrosystem. Environ Res Lett. 2016;11: 034004. doi: 10.1088/1748-9326/11/3/034004

[26] Varlamoff S, Florkowski WJ, Jordan JL, Latimer J, Braman K. Georgia Homeowner Survey of Landscape Management Practices. HortTechnology. 2001;11: 326–331. doi: 10.21273/HORTTECH.11.2.326

[27] Fleming M. Durham County Homeowner Fertilizer Behaviors Survey: Summary and Analysis of Results. 2013.

[28] Swann C. A survey of resident nutrient behavior in the Chesapeake Bay watershed. 1999.

[29] Rosen C, Horgan B, Mugaas R. Fertilizing Lawns. 2008. Available: https://hdl.handle.net/11299/200010

[30] Osmond DL, Platt JL. Characterization of Suburban Nitrogen Fertilizer and Water Use on Residential Turf in Cary, North Carolina. HortTechnology. 2000;10: 320–325. doi: 10.21273/HORTTECH.10.2.320

[31] Hadley S, Trechter D. Madison Area Municipal Storm Water Partnership: 2013 stormwater related perceptions, knowledge and practices survey report and 2014 online survey summary. 2014.

[32] Larsen L, Harlan SL. Desert dreamscapes: Residential landscape preference and behavior. Landsc Urban Plan. 2006;78: 85–100. doi: 10.1016/j.landurbplan.2005.06.002

[33] United States Geological Survey (USGS). Mineral Commodity Summaries 2022. 2022. Available: https://pubs.usgs.gov/periodicals/mcs2022/mcs2022-salt.pdf

[34] Kuemmel D, Hanbali R. Accident analysis of ice control operations. 1992.

[35] Upper Midwest Water Science Center. Evaluating chloride trends due to road-salt use and its impacts on water quality and aquatic organisms. United States Geological Survey; 2019. Available: https://www.usgs.gov/centers/upper-midwest-water-science-center/science/evaluating-chloride-trends-due-road-salt-use-and#overview

[36] Sparacino H, Stepenuck KF, Gould RK, Hurley SE. Review of Reduced Salt, Snow, and Ice Management Practices for Commercial Businesses. Transp Res Rec J Transp Res Board. 2021; 036119812110525. doi: 10.1177/03611981211052538

[37] Xiao D, Owusu-Ababio S, Schmitt R. Evaluation of the Effects of Deicers on Concrete Durability. 2018. Report No.: WHRP 0092-17-03. Available: https://rosap.ntl.bts.gov/view/dot/64318

[38] Hadley S, Trechter D. Rock River Stormwater Group: 2013 stormwater related perceptions, knowledge and practices survey report. 2014b.

[39] Public Sector Consultants. The use of selected deicing materials on Michigan roads: Environmental and economic impacts. 1993.

[40] U.S. Census Bureau QuickFacts: Hamilton County, Ohio. Www.census.gov. 2021. Available: https://www.census.gov/quickfacts/hamiltoncountyohio

[41] Meyer MH, Behe BK, Heilig J. The Economic Impact and Perceived Environmental Effect of Home Lawns in Minnesota. HortTechnology. 2001;11: 585–590. doi: 10.21273/HORTTECH.11.4.585

[42] Esri Inc. ArcGIS Pro. Esri Inc.; 2022. Available: https://www.esri.com/en us/arcgis/products/arcgis-pro/overview.

[43] Davis A, Herron O, Dumyahn S. Uncovering the potential for exurban properties and small working farms in the Midwestern United States to provide food and refuge for pollinators. Urban Ecosyst. 2021;24: 1047–1060. doi: 10.1007/s11252-021-01094-7

[44] Davis A, Stoyko J. Barriers to Native Plantings in Private Residential Yards. Land. 2022;12: 114. doi: 10.3390/land12010114

[45] Dillman D, Smyth J, Christian L. Internet, phone, mail, and mixed-mode surveys: the tailored design method. 4th ed. Wiley; 2014.

[46] Qualtrics. Provo, UT: Qualtrics; 2022. Available: https://www.qualtrics.com

[47] Murakami P, Oshiro K, Hensley D. Calculating the Amount of Fertilizer Needed for Your Lawn. 1999 [cited 7 Mar 2023]. Available: http://hdl.handle.net/10125/12597

[48] Hysell N. Identifying Optimal Ice Melt Application Rates for Greater Efficiency and Profits. In: Snow Magazine [Internet]. Sep 2011. Available: https://www.snowmagazineonline.com/article/snow-0911-identifying-optimal-ice-melt-application-rates-greater-efficiency-profits/

[49] Hossain S, Fu L, Lake R. Field evaluation of the performance of alternative deicers for winter maintenance of transportation facilities. Can J Civ Eng. 2015;42: 437–448.

[50] Hochmuth G, Trenholm L, Momod E, Rainey D, Lewis C, Bram N. The Role of Soil Management in Minimising Water and Nutrient Losses from the Urban Landscape. Jt Publ Mult Dep Programs UFIFAs. 2013.

[51] Raciti SM, Groffman PM, Jenkins JC, Pouyat RV, Fahey TJ, Pickett STA, et al. Accumulation of Carbon and Nitrogen in Residential Soils with Different Land-Use Histories. Ecosystems. 2011;14: 287–297. doi: 10.1007/s10021-010-9409-3

[52] Henry J, Gibeault V, Lazaneo V. Practical lawn fertilization. UCANR Publications; 2002.

[53] Environmental Protection Agency U.S. National management measures to control nonpoint source pollution from urban areas. U.S. Environ. Protection Agency, Washington, DC. 2005. Available: https://www.epa.gov/nps/national-management-measures-con trol-nonpoint-source-pollution-agriculture

[54] Carey RO, Hochmuth GJ, Martinez CJ, Boyer TH, Nair VD, Dukes MD, et al. A Review of Turfgrass Fertilizer Management Practices: Implications for Urban Water Quality. HortTechnology. 2012;22: 280–291. doi: 10.21273/HORTTECH.22.3.280

[55] Arthurs S, Stauderman K. Evaluating how accurately lawn fertilizers are applied using homeowner equipment. Proc Fla State Hortic Soc. 2010;123: 344–347.

[56] Nielson L, Smith CL. Influences on residential yard care and water quality: Tualatin watershed, Oregon. J Am Water Resour Assoc. 2005;41: 93–106. doi: 10.1111/j.1752-1688.2005.tb03720.x

[57] Cook E, Hall S, Larson K. Residential landscapes as social-ecological systems: a synthesis of multi-scalar interactions between people and their home environment. Urban Ecosyst. 2012;15: 19–52.

[58] Qian Y, Follett RF, Kimble JM. Soil Organic Carbon Input from Urban Turfgrasses. Soil Sci Soc Am J. 2010;74: 366–371. doi: 10.2136/sssaj2009.0075

[59] Cheng Z, Salminen S, Grewal P. Effect of organic fertilisers on the greening quality, shoot and root growth, and shoot nutrient and alkaloid contents of turf‐type endophytic tall fescue, Festuca arundinacea. Ann Appl Biol. 2010;156: 25–37.

[60] Sleeper F. Literature review: winter deicer maintenance practices on impervious surfaces: impacts on the environment. 2013.

[61] Budikova D, Ford T, Wright J. Characterizing winter season severity in the Midwest United States, part II: Interannual variability. Int J Climatol. 2022;42: 3499–3516.

[62] Nixon W, DeVries R. Development of a Handbook of Best Management Practices for Road Salt in Winter Maintenance Operations (No. CR14-10). 2015.

[63] Hintz WD, Fay L, Relyea RA. Road salts, human safety, and the rising salinity of our fresh waters. Front Ecol Environ. 2022;20: 22–30. doi: 10.1002/fee.2433

[64] Schindler DW. The dilemma of controlling cultural eutrophication of lakes. Proc R Soc B Biol Sci. 2012;279: 4322–4333. doi: 10.1098/rspb.2012.1032 22915669 PMC3479793

[65] Mazumder B, Wellen C, Kaltenecker G, Sorichetti RJ, Oswald CJ. Trends and legacy of freshwater salinization: untangling over 50 years of stream chloride monitoring. Environ Res Lett. 2021;16: 095001. doi: 10.1088/1748-9326/ac1817

[66] Michalak AM, Anderson EJ, Beletsky D, Boland S, Bosch NS, Bridgeman TB, et al. Record-setting algal bloom in Lake Erie caused by agricultural and meteorological trends consistent with expected future conditions. Proc Natl Acad Sci. 2013;110: 6448–6452. doi: 10.1073/pnas.1216006110 23576718 PMC3631662

[67] Watson SB, Miller C, Arhonditsis G, Boyer GL, Carmichael W, Charlton MN, et al. The re-eutrophication of Lake Erie: Harmful algal blooms and hypoxia. Harmful Algae. 2016;56: 44–66. doi: 10.1016/j.hal.2016.04.010 28073496

[68] Kollmuss A, Agyeman J. Mind the Gap: Why do people act environmentally and what are the barriers to pro-environmental behavior? Environ Educ Res. 2002;8: 239–260. doi: 10.1080/13504620220145401

[69] Liu P, Teng M, Han C. How does environmental knowledge translate into pro-environmental behaviors?: The mediating role of environmental attitudes and behavioral intentions. Sci Total Environ. 2020;728: 138126. doi: 10.1016/j.scitotenv.2020.138126 32361356

[70] Nassauer JI, Cooper DA, Marshall LL, Currie WS, Hutchins M, Brown DG. Parcel size related to household behaviors affecting carbon storage in exurban residential landscapes. Landsc Urban Plan. 2014;129: 55–64. doi: 10.1016/j.landurbplan.2014.05.007

[71] Robbins P. Lawn people: how grasses, weeds, and chemicals make us who we are. Temple University Press, Philadelphia; 2007.

[72] Law N, Band L, Grove M. Nitrogen input from residential lawn care practices in suburban watersheds in Baltimore county, MD. J Environ Plan Manag. 2004;47: 737–755. doi: 10.1080/0964056042000274452

[73] Campbell J, Rihn A, Khachatryan H. Factors Influencing Home Lawn Fertilizer Choice in the United States. HortTechnology. 2020;30: 296–305. doi: 10.21273/HORTTECH04454-19

[74] Wennberg H, Ståhl A, Hydén C. Older pedestrians’ perceptions of the outdoor environment in a year-round perspective. Eur J Ageing. 2009;6: 277–290. doi: 10.1007/s10433-009-0123-y 28798611 PMC5547343

[75] Rantakokko M, Iwarsson S, Portegijs E, Viljanen A, Rantanen T. Associations Between Environmental Characteristics and Life-Space Mobility in Community-Dwelling Older People. J Aging Health. 2015;27: 606–621. doi: 10.1177/0898264314555328 25326130

[76] Li Y, Hsu JA, Fernie G. Aging and the Use of Pedestrian Facilities in Winter—The Need for Improved Design and Better Technology. J Urban Health. 2013;90: 602–617. doi: 10.1007/s11524-012-9779-2 23188551 PMC3732686

