# Supplementary material for: Incongruence between residential uses and perceptions of fertilizers and deicers
Source: PLoS One. 2024 Jul 19;19(7):e0306550. doi: 10.1371/journal.pone.0306550 (PMC11259275; doi:10.1371/journal.pone.0306550)
Supplement: S1 Fig — (PDF) [file pone.0306550.s001.pdf]

MIAMI

Your Fertilizer and Deicer Use!

You are invited to participate in a research project being conducted by Heather Craska, a graduate student in the Institute for the Environment and Sustainability at Miami University. The purpose of this research is to **learn more about how you use fertilizers and deicers**. Participation in this research is **restricted to persons 18 years of age or older**.

The person who makes the lawn care decisions in your household should fill out the survey.

The survey should **8 minutes**. Your **participation is voluntary**, you may skip questions you do not want to answer, and you may stop at any time. The survey will not ask for information about your identity. If you inadvertently include identifying information, such information will be removed from any stored data. Only the research team will have access to individual responses. Results of the research will be presented publicly only as aggregate summaries.

If you have any questions about this research or you feel you need more information to complete this survey, you can **contact me at** craskahd@miamioh.edu or Dr. Amelie Davis at davis.amelie@miamioh.edu. If you have questions or concerns about the rights of research subjects, you may contact our reviewing body: **Research Ethics and Integrity Office at Miami University at (513) 529-3600 or humansubjects@miamioh.edu.**

PLEASE START SURVEY HERE

Q1. Do you or someone in your household maintain your own lawn?

- □ Yes
- □ No
- □ I'm not sure

Q2. Do you or someone in your household maintain your own sidewalks and driveway?

- □ Yes
- □ No
- □ I'm not sure

Q3. Do you belong to a homeowner's association (HOA)?

- □ Yes
- □ No
- □ I'm not sure

Q4.If you answered yes in Q3, does this HOA have strict lawn maintenance guidelines?

- □ Yes
- □ No
- □ I'm not sure

Q5. If you answered yes in Q3, does this HOA require you to clear snow and ice from the following areas?

Sidewalks: □ Yes □ No □ I'm not sure

Driveways: □ Yes □ No □ I'm not sure

This research can only be successful with your help.

Your Fertilizer Use

Q6. Do you or someone else apply fertilizer to your property at least once a year? (If you answer no, please skip to Q19 under "Your Deicer Use".) □ Yes □ No □ I do not know

If NO, skip to Q19 on next page

Q7.During which month(s) of the year do you or someone else apply fertilizers to your property? (Check all that apply)

□ January □ February □ March □ April □ May □ June
□ July □ August □ September □ October □ November □ December

Q8. How often do you or someone else add fertilizer to your lawn or garden?

□ Once a year
□ More than once a month
□ Every few months
□ Not sure
□ Monthly

Q9.While you have lived on this property, have you ever tested your soil (for pH and nutrients)?

□ Yes □ No □ I do not know

Q10. If you answered yes in Q9., do you test your soil yearly?

□ Yes □ No □ I do not know

Q11. Do you use granular or liquid fertilizer on your lawn? □ Granular □ Liquid □ Both

Q12. Approximately how much fertilizer do you apply to your property EACH YEAR?

I use (#)_________ bags that each weigh about________ pounds **(GRANULAR)**
I use (#)_________ containers that each contain about________ fl oz **(LIQUID)**

Q13. Do you or someone else apply similar amounts of fertilizer to all areas of your lawn (e.g. front yard, back yard, garden)? □ Yes □ No □ I do not know

Q14. If you answered no in Q13, please explain which areas of your property receive larger or smaller amounts of fertilizer.

____

Q15. Why do you apply fertilizer to your lawn? (Check all that apply and circle the main reason)

□ To maintain the appearance of my property
□ To support plant growth and health
□ To meet HOA regulations
□ It is the neighborly thing to do
□ I am not sure
□ Other (please specify):_________________________________

Q16. Do you or someone else sweep excess fertilizer off driveways, sidewalks, or the road back into the lawn or garden?

□ All of the time
□ I am not sure
□ Sometimes
□ Other (please specify):______________________
□ Never

Q17. What method do you/ a contractor use to apply fertilizer to your lawn?

□ Drop Spreader
□ Spread by Hand
□ Broadcast Spreader
□ I am not sure
□ Hand Spreader
□ Other (please specify):______________________

Q18. If YOU apply your own fertilizer, how do you decide how much fertilizer to apply?

____

This research can only be successful with your help.

Your Deicer Use

Q19. Do you or someone else apply deicers (rock salt / ice melt) to your property at least once a year? (If you answer no, please skip to Q28) □ Yes □ No □ I do not know

If NO, skip to Q28 on next page

Q20. During which month(s) of the year do you or someone else apply deicers to your property? (Check all that apply)

□ January □ February □ March □ April □ May □ June
□ July □ August □ September □ October □ November □ December

Q21. What prompts you or someone else to apply deicers to your property during the times of year when you use them? (Check all that apply)

□ When I see a weather forecast predicting ice/snow accumulation
□ When I see a weather forecast predicting freezing temperatures
□ After snow has already accumulated
□ After ice has already accumulated
□ Other____
□ I am not sure

Q22. When you do use deicers, which of the following areas on your property receive deicer?

**Driveway:**	□ Never	□ Rarely	□ Sometimes	□ Often	□ Always	□ Not Applicable
**Sidewalk:**	□ Never	□ Rarely	□ Sometimes	□ Often	□ Always	□ Not Applicable
**Front steps/porch:**	□ Never	□ Rarely	□ Sometimes	□ Often	□ Always	□ Not Applicable
**Patio:**	□ Never	□ Rarely	□ Sometimes	□ Often	□ Always	□ Not Applicable
**Lawn:**	□ Never	□ Rarely	□ Sometimes	□ Often	□ Always	□ Not Applicable
**Other**___________ :	□ Never	□ Rarely	□ Sometimes	□ Often	□ Always	□ Not Applicable

Q23. For the areas you selected in Q22., do you apply deicer to the entire area or only the parts with ice / snow accumulation?

**Driveway:**	□ The entire area	□ Only the parts with ice/snow	□ I don't apply salt here
**Sidewalk:**	□ The entire area	□ Only the parts with ice/snow	□ I don't apply salt here
**Front steps/porch:**	□ The entire area	□ Only the parts with ice/snow	□ I don't apply salt here
**Patio:**	□ The entire area	□ Only the parts with ice/snow	□ I don't apply salt here
**Lawn:**	□ The entire area	□ Only the parts with ice/snow	□ I don't apply salt here
**Other:**______________	□ The entire area	□ Only the parts with ice/snow	□ I don't apply salt here

Q24. What kinds of deicers do you or someone else use on your property? (Check all that apply)

□ Chloride-Based Deicers (Sodium Chloride, Calcium Chloride, Magnesium Chloride)
□ Acetate-Based Deicers (Calcium magnesium acetate, Potassium acetate, Sodium acetate)
□ Carbohydrate Deicers (Beet Juice, Molasses, Corn Syrup)
□ Safe Paw (or other non-salt deicers marketed for pet safety)
□ I am not sure
□ Other____

Q25. Approximately how much deicer do you apply to your property over the course of a year?

I use (#)_________ bags that each weigh about________ pounds

Q26. How do you decide how much deicer to apply to your property?

____

This research can only be successful with your help.

Q27. Why do you apply deicers to your property? (Check all that apply and circle the main reason)

- □ Safety Reasons
- □ It is the neighborly thing to do
- □ To avoid liability from ice related injuries on my property
- □ To be able to move my vehicles to and from my driveway
- □ To make sidewalks and pathways easier to walk on
- □ To make my property more visually appealing
- □ To prevent ice related damages to my property
- □ Other (please specify):_______________________________

Q28. **Please check the box that most accurately describes your opinion towards each statement.**

Please check the most appropriate box	Strongly Disagree	Disagree	Agree nor Disagree	Agree	Strongly Agree	Not Applicable
It is **important** for me to have a **lush, green lawn.**	☐	☐	☐	☐	☐	☐
I take into **consideration** how **my lawn care practices might affect the environment** when applying fertilizer.	☐	☐	☐	☐	☐	☐
Having an **attractive lawn reflects positively** on me as a **resident** in my neighborhood.	☐	☐	☐	☐	☐	☐
**Using fertilizers** to have an attractive lawn is the **neighborly thing to do.**	☐	☐	☐	☐	☐	☐
**I use** fertilizers in a way that **minimizes** negative **environmental effects.**	☐	☐	☐	☐	☐	☐
I am **concerned** about **myself**, my **family**, **or my pets** being exposed to the **chemicals** in **fertilizers**.	☐	☐	☐	☐	☐	☐
I am **interested in learning** more about how to **properly** and **safely apply fertilizers** to my property.	☐	☐	☐	☐	☐	☐
In the future, I will consider making **fertilizer choices** which are **safer** for the **environment.**	☐	☐	☐	☐	☐	☐

Please check the most appropriate box	Strongly Disagree	Disagree	Agree nor Disagree	Agree	Strongly Agree	Not Applicable
It is **important** for me to remove and/or prevent ice accumulation on my property.	☐	☐	☐	☐	☐	☐
I take into **consideration** how **my deicer use might affect the environment**.	☐	☐	☐	☐	☐	☐
**I use deicers** on my property because I am **concerned** about the **safety** of my **family and myself.**	☐	☐	☐	☐	☐	☐
Removing ice from my property **reflects positively** on me as a **resident** in my neighborhood.	☐	☐	☐	☐	☐	☐
**I use deicers** on my property because it is the **neighborly thing to do.**	☐	☐	☐	☐	☐	☐
**Deicers** contribute to negative **environmental impacts.**	☐	☐	☐	☐	☐	☐
I use deicers in a way which **reduces** negative **environmental impacts.**	☐	☐	☐	☐	☐	☐
I am concerned about **myself, my family or my pets** being exposed to the **chemicals** in **deicers**.	☐	☐	☐	☐	☐	☐
I am **interested in learning** more about how to properly and **safely apply deicers** to my property.	☐	☐	☐	☐	☐	☐
In the future, I will consider making **deicer choices** which are **safer** for the **environment.**	☐	☐	☐	☐	☐	☐

Last page next!

BACKGROUND INFORMATION

This section is for statistical purposes only.

As a reminder, all survey responses will be strictly confidential.

Do pets play on or use your lawn? ☐ Yes ☐ No

Do children play on or use your lawn? ☐ Yes ☐ No

How would you classify your property type? ☐ Rural ☐ Suburban ☐ Urban

What is the employment status of the main earner in your household?

☐ Full-time ☐ Part-time ☐ Not working ☐ Retired ☐ Student

What is the highest level of education you have completed?

☐ Some High School ☐ High School/GED ☐ Certificate Program

☐ Associates Degree ☐ Some College ☐ 4-year College Degree

☐ Master's Degree ☐ Doctoral Degree (PhD, JD, MD)

How old are you? ______ years

What is your gender? ☐ Male ☐ Female

How do you identify your race/ethnicity?

☐ White ☐ Hispanic ☐ African American ☐ American Indian

☐ Asian/Pacific Islander ☐ Other

What was your total household income last year, before taxes? Please check ONE answer

☐ Less than $25,000 ☐ $100,000 up to $150,000

☐ $25,000 up to $50,000 ☐ More than $150,000

☐ $50,000 up to $100,000

Do you have any final comments you would like to share with us?

____

____

____

____

____

Thank you greatly for completing our survey!

Your participation is very important to us and we value the information you provide.

If you wish to return this survey by mail, please mail it to Dr. Amelie Davis, Department of Geography, Miami University, 250 S. Patterson Ave, Oxford OH, 45056.
