# Supplementary figures and images for: Incongruence between residential uses and perceptions of fertilizers and deicers
Source: PLoS One. 2024 Jul 19;19(7):e0306550. doi: 10.1371/journal.pone.0306550 (PMC11259275; doi:10.1371/journal.pone.0306550)

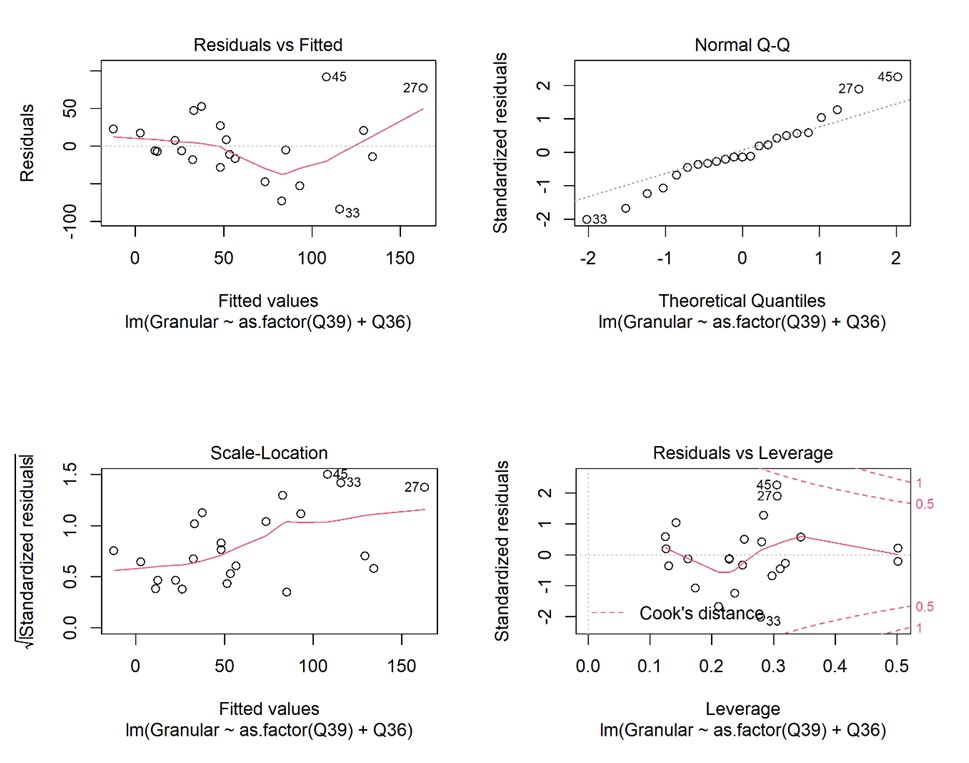

Supplement: S2 Fig — (TIF) [file pone.0306550.s002.tif]
